# Development of a 3D angiogenesis model to study tumour – endothelial cell interactions and the effects of anti-angiogenic drugs

**DOI:** 10.1038/s41598-017-03010-6

**Published:** 2017-06-07

**Authors:** Arno Amann, Marit Zwierzina, Stefan Koeck, Gabriele Gamerith, Elisabeth Pechriggl, Julia M. Huber, Edith Lorenz, Jens M. Kelm, Wolfgang Hilbe, Heinz Zwierzina, Johann Kern

**Affiliations:** 1 Medical University of Innsbruck, Department of Internal Medicine V, Anichstraße 35, 6020 Innsbruck, Austria; 2 Medical University of Innsbruck, Department of Plastic, Reconstructive and Aesthetic surgery, Anichstraße 35, 6020 Innsbruck, Austria; 3 Medical University of Innsbruck, Division of Clinical and Functional Anatomy, Department of Anatomy, Histology and Embryology, Müllerstraße 59, 6020 Innsbruck, Austria; 4 InSphero AG, Wagistrasse 27, CH-8952 Schlieren, Switzerland; 5 Wilhelminenspital, Medical Department, Centre for Oncology, Haematology and Palliative care, Motlearstraße 37, 1160 Vienna, Austria; 6 Tyrolean Cancer Research Institute, Innrain 66, 6020 Innsbruck, Austria

## Abstract

The tumour microenvironment and tumour angiogenesis play a critical role in the development and therapy of many cancers, but *in vitro* models reflecting these circumstances are rare. In this study, we describe the development of a novel tri-culture model, using non-small cell lung cancer (NSCLC) cell lines (A549 and Colo699) in combination with a fibroblast cell line (SV 80) and two different endothelial cell lines in a hanging drop technology. Endothelial cells aggregated either in small colonies in Colo699 containing microtissues or in tube like structures mainly in the stromal compartment of microtissues containing A549. An up-regulation of hypoxia and vimentin, ASMA and a downregulation of E-cadherin were observed in co- and tri-cultures compared to monocultures. Furthermore, a morphological alteration of A549 tumour cells resembling “signet ring cells” was observed in tri-cultures. The secretion of proangiogenic growth factors like vascular endothelial growth factor (VEGF) was measured in supernatants. Inhibition of these proangiogenic factors by using antiangiogenic drugs (bevacizumab and nindetanib) led to a significant decrease in migration of endothelial cells into microtissues. We demonstrate that our method is a promising tool for the generation of multicellular tumour microtissues and reflects *in vivo* conditions closer than 2D cell culture.

## Introduction

Different molecular processes lead to metastatic spread and the occurrence of tumour cell resistance to therapeutic interventions. Among them, lately the crucial role of tumour stroma inducing drug resistance by secretion of growth hormones and cytokines has been identified^1–3^.

So far, many of these paracrine activators have been evaluated as potential drug candidates. Vascular endothelial growth factor (VEGF), its target receptor and the associated complex process of tumour angiogenesis has been proven to be a promising target for research and for the effective treatment of cancer patients^4, 5^.

During tumour growth, oxygen and nutrient deprivation arises not only in the tumour but also in the surrounding tissues, triggering the release of angiogenic growth factors and cytokines such as vascular endothelial growth factor (VEGF), fibroblast growth factor (FGF), platelet derived growth factor (PDGF) and interleukin-8 (IL-8). These factors stimulate endothelial and perivascular cells in the neighbourhood, leading either to the generation of new vessels or the recruitment of surrounding vessels in normal tissues^6^. As a result, the effort of developing angiogenic inhibitors for these molecular targets led to the clinical development of a variety of anti angiogenic drugs for cancer treatment.

The monoclonal antibody bevacizumab was tested in clinical trials in most solid tumours and only achieved an approval for treatment of colorectal cancer, renal cell carcinoma and breast cancer^7^. However, in the majority of patients the efficacy has been proven to only be of rather short duration. One reason for the limited duration of response is the tumours’ ability to overcome VEGF blockade by the activation of salvage pathways leading either to neo-vasculogenesis, vascular mimicry, vessel co-option or to remodelling of neighbouring blood vessels^7^.

Furthermore, the lack of predictive markers that help to define patient subgroups that benefit most from an anti-VEGF therapy prevents us from developing more tailored treatment modalities. Predictive markers are also still missing for other novel anti-angiogenic drugs^8, 9^.

Therefore, novel *in vitro* models are needed mimicking angiogenesis and resemble more closely the *in vivo* conditions.

So far, substances have been initially tested predominantly in cell culture flasks. Being effective in this model, substances were tested in mice and then further investigated in clinical trials. Despite positive phase I/II data, in recent years more and more drugs failed in phase III trials due to the lack of efficacy in large cohorts of patients. Especially targeted therapies “proved to be a graveyard for research money in oncology”^10^.

So far cell-based assays to explore cell biology and drug efficacy were performed on two-dimensional plastic surfaces or in single cell suspension. The biology of cells, however, being profoundly influenced by their micro-environment, require cell based assays that reflect the effects of factors such as the extracellular matrix (ECM), cell-cell contacts, cell-matrix interactions, cell polarity and oxygen profiles^11–13^.

Conventional two dimensional (2D) cell culture systems, where cells grow on artificial plastic surfaces fail to adequately represent the mentioned interactions. In contrast, 3D cell cultures offer a way to cultivate cells in a more sophisticated environment where ECM and cell-cell contacts can be mimicked.

The hanging drop technique is a well-established cell culture method to form spherical microtissues from immortalized and primary cell lines^11, 13–16^.

In contrast to most liquid overlay technologies, tissue engineered models or microfluidic devices, the hanging drop model allows the precise control over the initial cell population in each microtissue. It also enables the addition of new cells, drugs and media at any time to reach a long term cultivation of cells and drugs can be realized. Furthermore, it enables the generation of high numbers of reproducible microtissues and thus makes it possible to test drugs in a standardized fashion.

Neither additional supplements nor artificial scaffolds mimicking extracellular matrix components (e.g. collagen matrigel) are required to generate multi-cell type co-culture microtissues.

Based on an automation and high-throughput compatible hanging drop technology we improved our existing organotypic co-culture models composed of two different non-small cell lung cancer (NSCLC) cell lines in combination with lung fibroblasts, by adding two endothelial cell lines to generate an even more *in vivo* like cell culture model. This novel model allows us to investigate tumour-stroma interactions in combination with endothelial cells in a model were cell growing is not influenced by artificial ECM to grow. Furthermore, it represents a 3D angiogenesis model where targeted anti-angiogenic therapies can be tested in a large and reproducible way.

## Results

### Cell organization and protein expression pattern in multicellular microtissues

**Figure 1:**
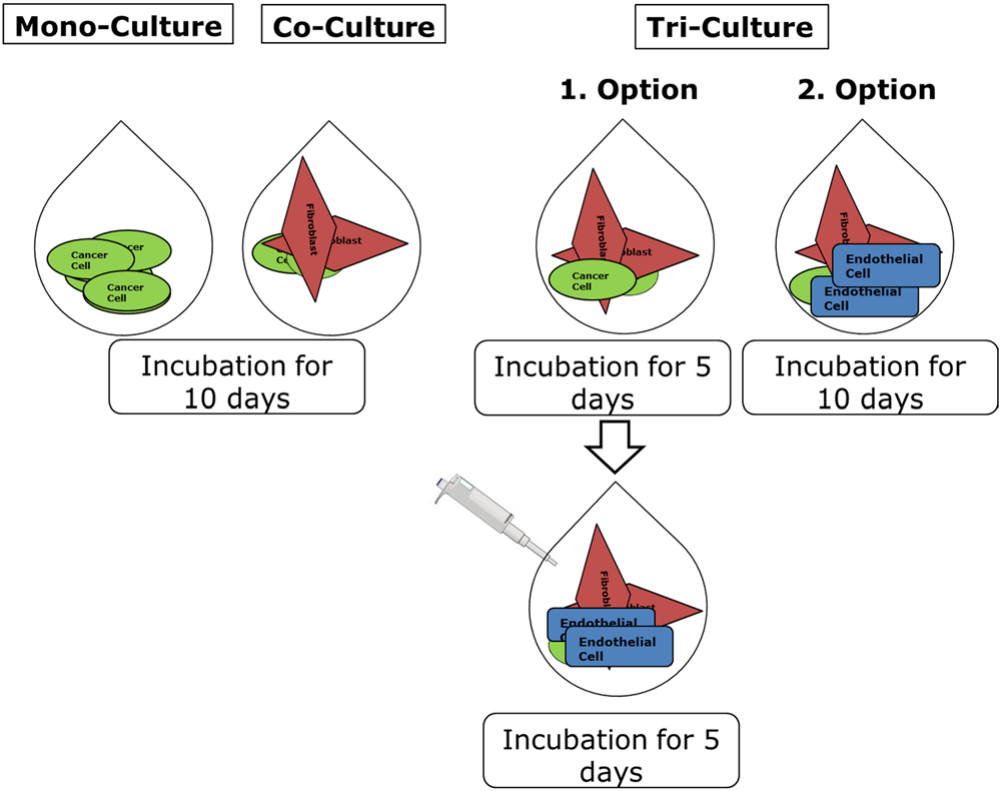
Systematic picture of how cells were seeded in hanging drops either as mono-, co- or tri-cultures. Endothelial cells were seeded in two ways together with cancer cells and fibroblasts. Option 1: endothelial cells were added as a mixture of single cells together with fibroblasts and cancer cells for an incubation time of ten days; Option 2: endothelial cells were added to pre-incubated co-cultures of tumour cells and fibroblasts on day five for another five days before all of them were harvested on day ten.

Immunohistochemistry was performed to analyse the architecture of microtissues and the expression of proteins (Fig. 1 + Supplementary Table 2). Protein expression was compared to the results of mono and co-cultures, which were already published by our working group^11^.

**Figure 2:**
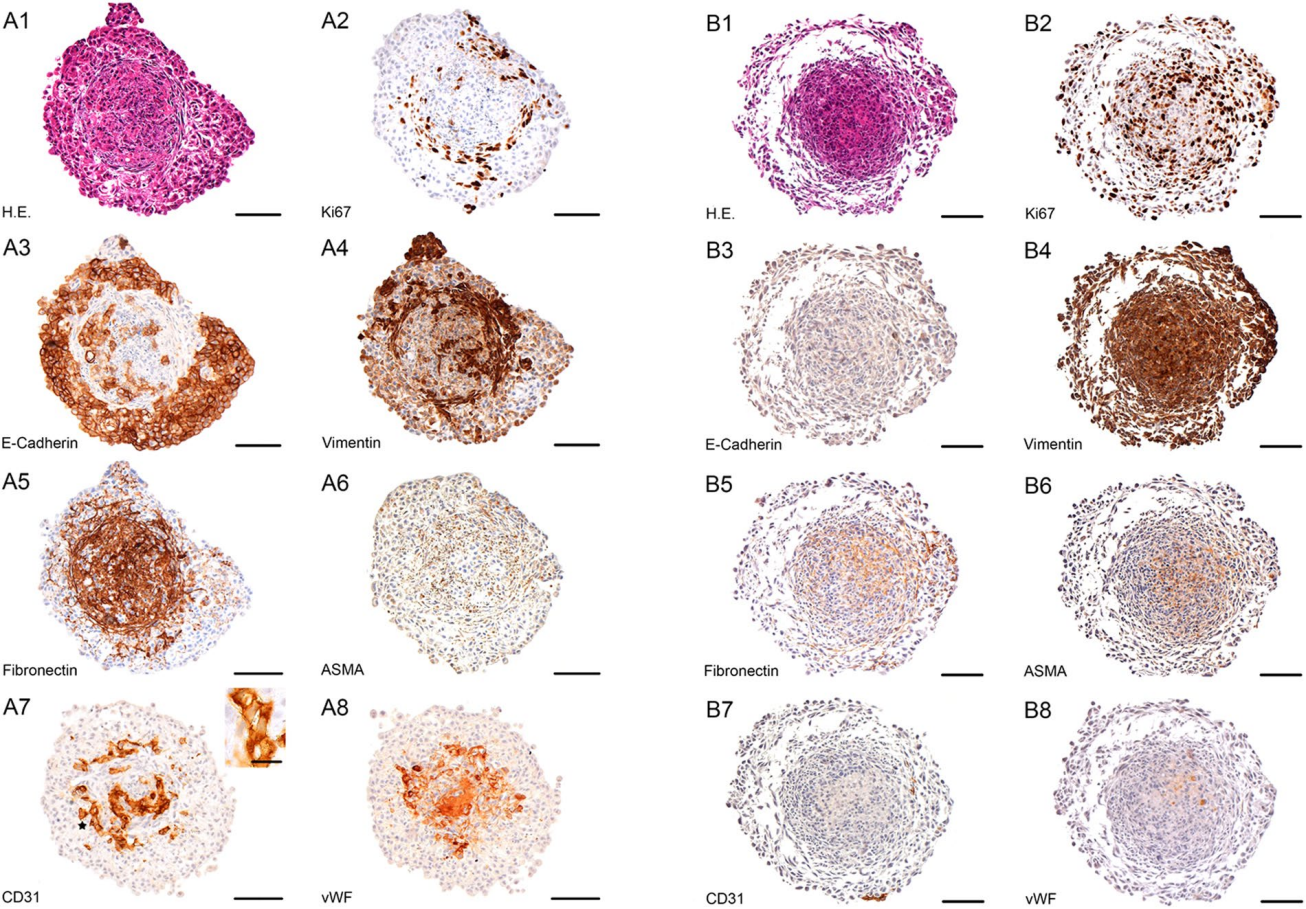
(**A**) A549 tri-cultures microtissue protein expression pattern (A1–A8): IHC slices of A549 with SV80 and HUVECs after 10 days. Endothelial cells are located in the fibroblast core of microtissues (A7). All cells started to express alpha-smooth muscle actin (A7) and vimentin (A4). Bar: 100 µm. Inlet showing formation of endothelial cells as coherent structure. Bar: 20 µm. (**B**) Colo699 tri-cultures microtissue protein expression pattern (B1–B8): IHC slices of Colo699 with SV80 and HUVEC after 10 days. Endothelial cells aggregated in small isles at the surface of microtissues (B7). All cells expressed ASMA (B6), vimentin (B4) and no E-cadherin (B3). Bar: 100 µm.

Vimentin and ASMA are applied as markers for mesenchymal or transformed cells, in our case of cancer associated fibroblasts or transformed tumour cells due to epithelial to mesenchymal transition (Fig. 2A,B).

E-cadherin was used to highlight areas within the microtissues, which are enriched by epithelial cancer cells (Fig. 2A,B). Endothelial cells were identified by the expression of CD31 and vWF staining was performed to visualize their functional ability to produce clotting enzymes (Fig. 2A,B). When endothelial cells were seeded together with fibroblasts and tumour cells on day one, no endothelial cells were located in the microtissues (data not shown).

**Figure 3:**
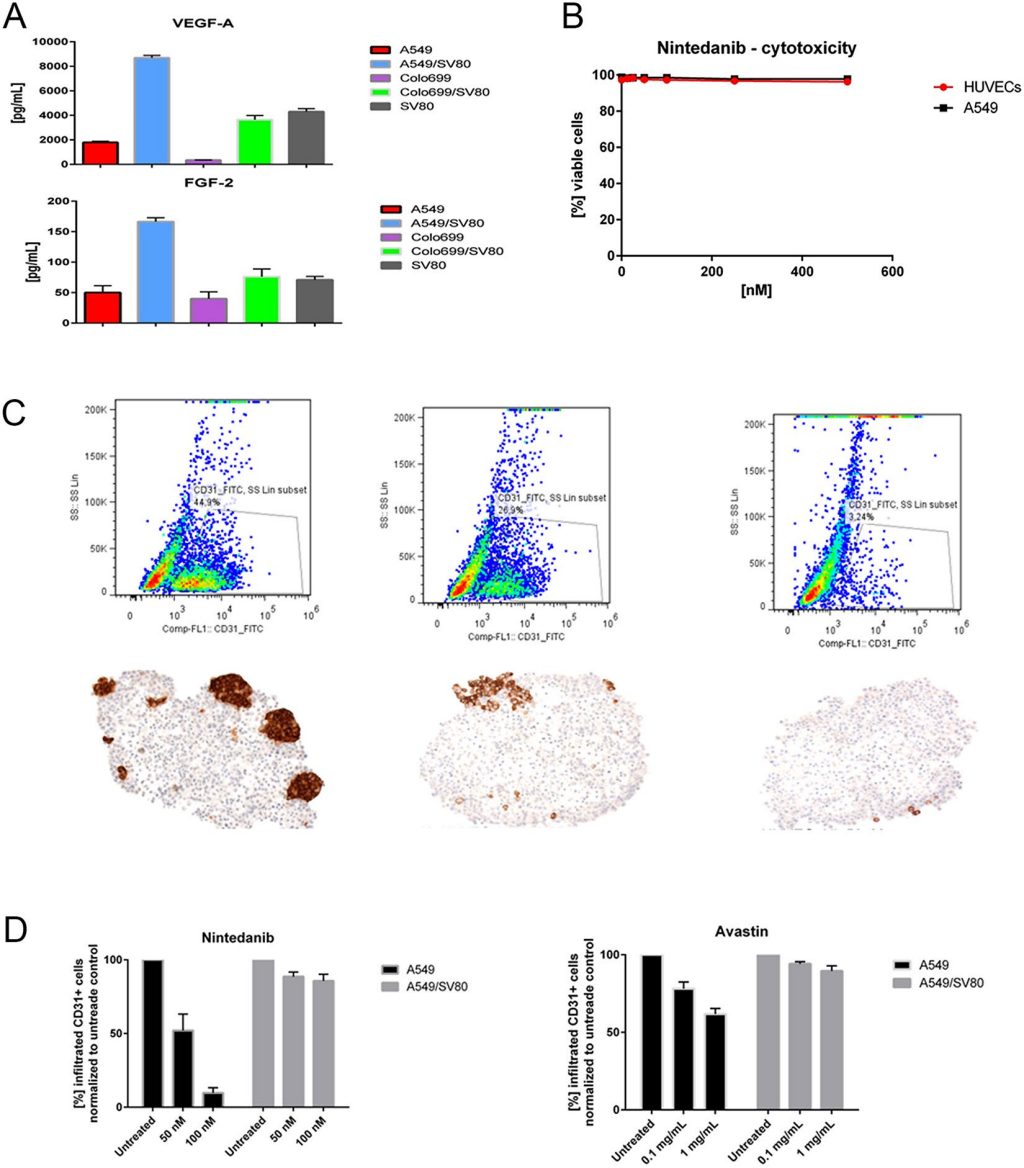
Cytokine secretion profile and anti angiogenic drug incubation: (**A**,**B**) Supernatants from monocultures (A549, Colo699, SV80) and co-cultures (A549/SV80), Colo699/SV80) were harvested on the day endothelial cells were added and analysed by a multiplex immunoassay (ProcartaPlex Human Growth Factor Panel 11 plex” – immunoassay). Co-cultures of cancer cells with fibroblasts delivered high amounts of pro-angiogenic factors (vascular endothelial growth factor (VEGF) and fibroblast growth factor (FGF)). (**C**) Dose depending cytotoxicity of nindetanib on cancer cells and endothelial cells. No dose depending cytotoxicity was measured for both cell lines. D + E Blocking endothelial cell migration into microtissues by inhibition of angiogenesis. Either bevacizumab or nindetanib were added to tri-cultures for 48 hours. Migration of endothelial cells into microtissues was displayed either by flow cytometry or IHC. Incubation with the two substances led to a decrease or a complete stop in migration of endothelial cells. A dose depending effect on the inhibition of endothelial cell migration was observed.

Co-cultures were pre-incubated for five days to guarantee the secretion of pro-angiogenic (VEGF, FGF) factors into the supernatant (Fig. 3).

In all tri-cultures containing A549, discrimination between E-cadherin expressing tumour and vimentin expressing mesenchymal cells was performed after ten days. Tumour cells expressing E-cadherin were located either in small cell isles in the outer parts of microtissues or in the inner core of vimentin expressing cells. These cells were displaying either a strong vimentin or CD31 expression and thus were defined to be fibroblasts or endothelial cells. All CD31 positive cells were located either in or around accumulations of vimentin expressing mesenchymal cells near the core of microtissues. Furthermore, CD31+ cells formed adherent colonies mimicking tube like structures (Inlet Fig. 2A).

**Figure 4:**
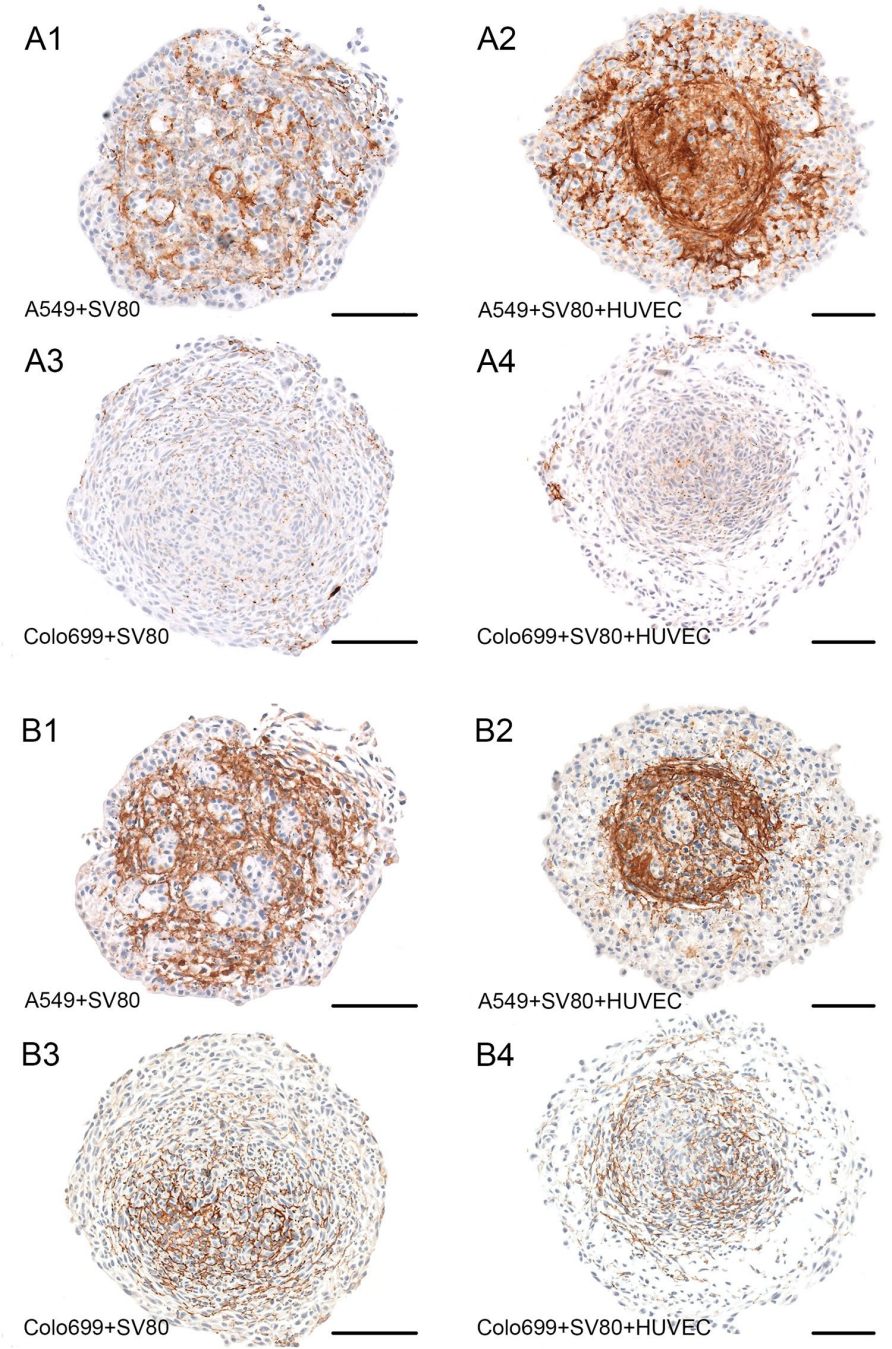
Production of ECM. Fibronectin A1–A4, Collagen VI B1–B4, Bar: 100 µm. Production of Fibronectin and Collagen VI was observed in all microtissues that consisted of both cancer cells and stromal cells. High secretion of ECM was displayed around the core and in areas where stromal cells predominantly formed the microtissue.

Nearly no CD31 positive cells were found in the E-cadherin positive areas of the spheroid. Fibronectin was also expressed especially in the core of microtissues, allocated as a ring around the core bordering to E-cadherin positive cells. In addition to Fibronectin, Collagen VI expression was investigated to further highlight the generation of ECM in our microtissue. Collagen VI was found to be significantly expressed in all microtissues that included fibroblasts and A549 cancer cells but not in Colo699 containing microtissues (Fig. 4).

In general, no difference in protein expression patterns were observed between either HUVECs or L-HMVECs containing microtissues (Supplementary Figs 1 and 2, Supplementary Table 2).

In both tri-cultures, all cells expressed vimentin and Ki-67. In contrast to tri-cultures containing A549 cells, endothelial cells, indicated by a positive CD31 and vWF pattern, were only very rarely found in the outer part of the microtissue. Most endothelial cells adhered to themselves in small colonies either next to the microtissue surface or as loose small spheres at the outer rim of the microtissue.

### Migration of endothelial cells and formation of adherent CD31+ tube-like structures

**Figure 5:**
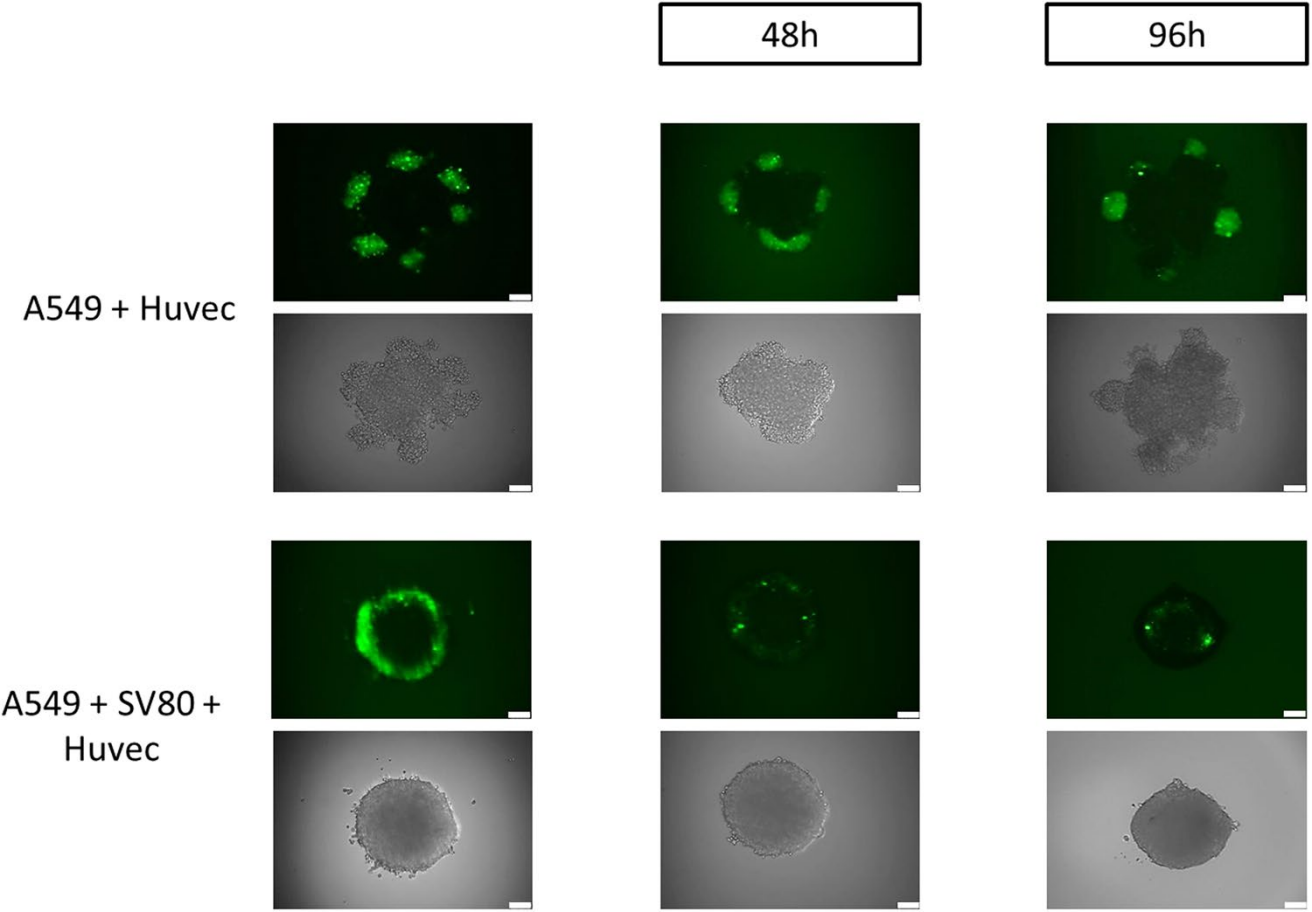
Migration of endothelial cells in A549 containing microtissues. Endothelial cells were labelled with CFSE and cells attached after 24 hours to the microtissue and migrated during cultivation time to the core of the spheroids.

In addition to CD31, CFSE labelled endothelial cells were tracked by daily imaging with an epifluorescent microscope to show migration of endothelial cells into preformed microtissues (Fig. 5).

A time dependant adhesion and migration of endothelial cells was observed until 96 hours after adding them to mono- and co-cultures.

However, endothelial cells displayed significantly higher migration ability into microtissues containing both cancer cells and fibroblasts.

No significant difference in migration of endothelial cells was observed between microtissues containing either Colo699 or A549 cancer cells (Fig. 5, Supplementary Fig. 3).

### Tumour stroma interactions lead to a transformation of tumour cells and fibroblasts

ASMA, a protein that is not expressed in regular fibroblasts, was applied as a marker that indicated a transformation of fibroblasts to so called cancer associated fibroblasts. In addition, staining for ASMA in combination with vimentin was used to define transformed tumour cells as a sign of epithelial to mesenchymal transition (EMT) (Fig. 2A,B, Supplementary Table 2) since ASMA is not expressed in 3D monocultures of cancer cells.

When A549 cells were included in the microtissues, ASMA and vimentin expression was found throughout the whole microtissue in all cells (fibroblasts and endothelial cells).

This expression pattern could also be shown in Colo699 microtissues, where ASMA was expressed in all displayed cells.

**Figure 6:**
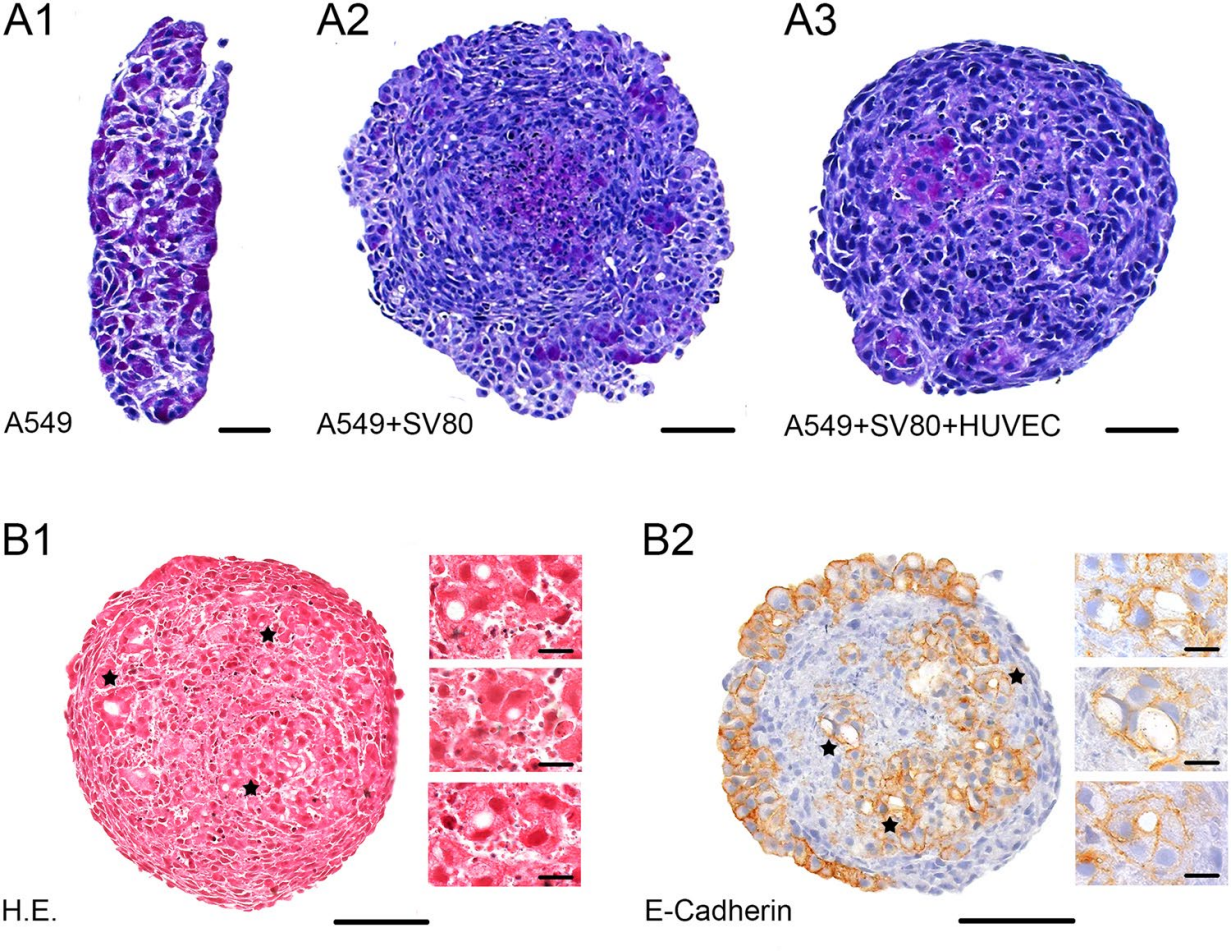
Glycoprotein expression and morphologic alteration of cancer cells. A1–A3: Periodic acid Schiff stain (PAS) positive cells increased in tri-cultures. Bar: 50 µm. B1 + B2: Furthermore, A549 cancer cells started to resemble so-called signet ring cells. Asteriks indicates the localization of the insert. Bar: 100 µm, Inserts 15 µm.

Periodic acid Schiff stain (PAS) was used to investigate whether cancer cells are able to produce glycoproteins (Fig. 6). Compared to monocultures, co- and tri-cultures of A549 containing microtissues showed an increase in PAS positive cells. PAS positive areas of cells were found to be larger only in microtissues where endothelial cells were included (Fig. 6).

Interestingly, in these microtissues, E-cadherin positive cells (only A549) started to display an altered morphology resembling so called signet ring cells. These alterations are known to be strong indicators for a more aggressive phenotype.

In patients these cells can be found sometimes in NSCLC, gastric or colorectal cancer patients.

### Viability of endothelial cells in co-cultures

**Figure 7:**
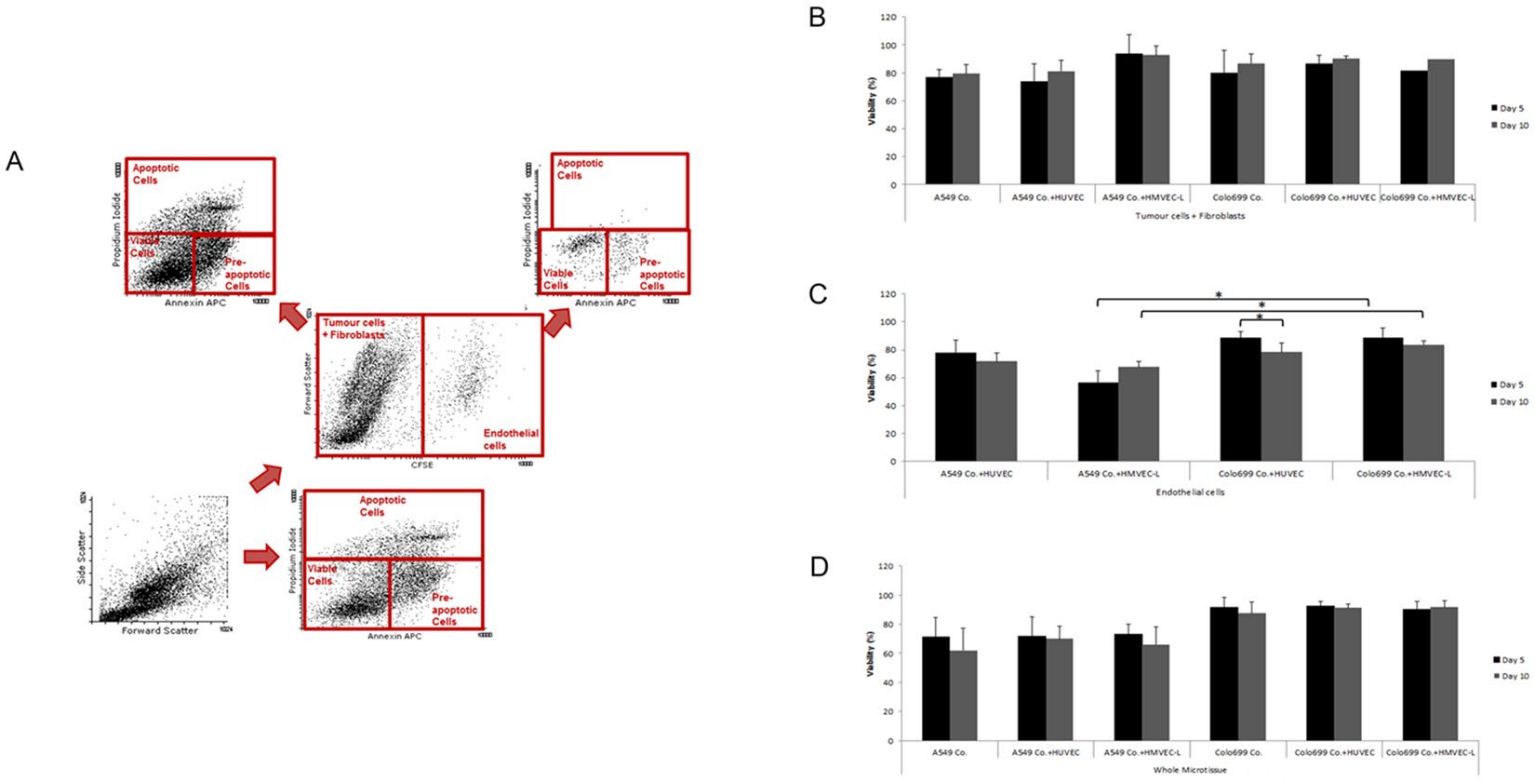
Viability of microtissues: Viability was measured with an Annexin V and Propidium Iodide protocol for flow cytometry. Apoptosis was measured after ten days. Each bar represents the mean cell viability of 18 spheroids and their corresponding standard deviation that was measured in two different runs. Viability in all microtissues was not altered when co-incubated with endothelial cells. Nevertheless, endothelial cells also remained viable throughout the whole incubation period. No difference was observed between the two different endothelial or cancer cell lines.

To determine cell viability, an Annexin V APC and Propidium Iodide (PI) FACS assay was performed (Fig. 7).

When endothelial cells were cultivated alone, no microtissues developed during the whole incubation period, due to an apoptotic rate of 100% in the first 72 hours (data not shown).

In contrast to monocultures, endothelial cells remained viable during the whole incubation period when co-cultivated with cancer cell lines and fibroblasts. In HUVEC containing microtissues, 78% after five days and 72% after ten days were found to be viable while an increase in viability was observed in L-HMVEC co-cultures with viable endothelial cells ranging from 56% after five to 68% after ten days of incubation.

In Colo699 and SV80 co-cultures the average viability of all endothelial cells remained higher during the incubation time. A significant increase in viability of L-HMVEC cells was observed compared to A549/SV80 co-cultures, ranging from 89% after five days to 84% after ten days (p > 0,05). In addition, HUVECs in combination with Colo699 and fibroblasts displayed also an increase in viable cells compared to other tri-cultures, displaying 89% viable cells after five and 79% after ten days.

When the viability of the tumour cell/fibroblast fraction was analysed and compared, an increase of viable cells was determined in tri-cultures containing L-HMVEC. The percentage of viable cells remained stable at 93% after five and ten days compared to 80% and 81% of viable cells in tri-cultures of A549 cells and HUVECs and A549/SV80 co-cultures.

### Cell proliferation and activation in microtissues

Ki-67 expression was analysed by means of IHC. Results of tri-cultures were also compared to already published data of the IHC pattern of mono- and co-cultures^11^ (Supplementary Fig. 4).

All Ki-67 results that are shown only refer to tri-cultures where endothelial cells were added after five days of tumour/fibroblast co-cultures.

In tri-cultures consisting of A549 and HUVECs, 75% of all cells were stained positive for Ki-67. Microtissues with L-HMVECs showed significantly fewer cells positive for this marker, counting up to only 16% of all cells (p < 0,001). Similar results were found in Colo699 consisting microtissues. In HUVECs containing microtissues, 98% of all cells were positive for Ki-67 compared to only 70% positive cells with L-HMVECs. This was found to be statistically significant (p < 0,05).

### Oxygen deprivation and hypoxia

Oxygen deprivation was analysed by the expression of carbonic anhydrase 9 (CA IX) since the diameter of our microtissues extended beyond 250 µm. At this diameter, hypoxia starts to play a crucial role due to critical reduction of oxygen diffusion.

**Figure 8:**
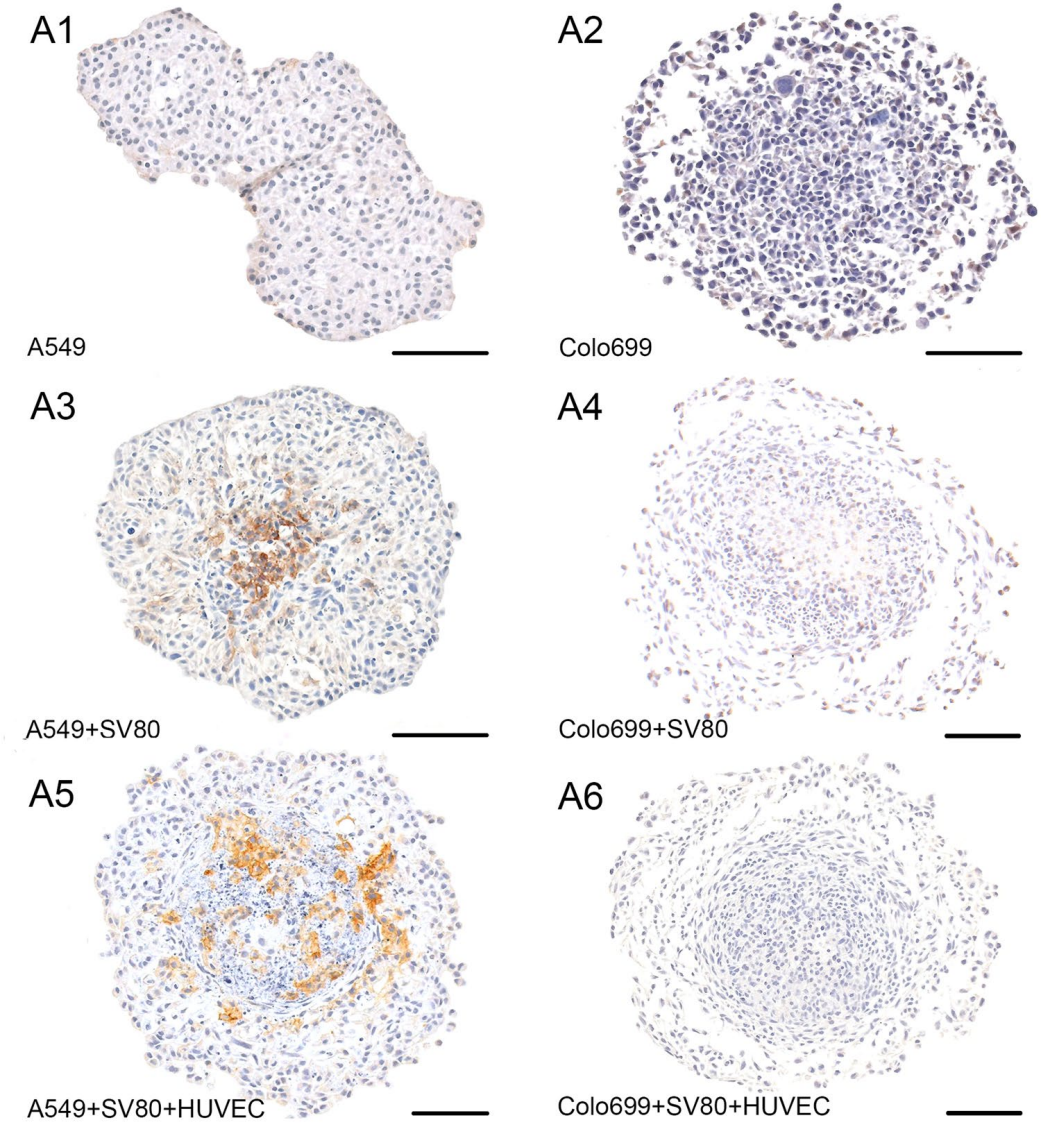
Oxygen deprivation leading to hypoxia: Carboanhydrase IX (CA IX) was analysed to identify hypoxia in ten day old microtissues (A1–6). This enzyme was only expressed in microtissues that consisted not only of cancer cells but also of the stromal compartment (A3–6). However, CA IX was secreted predominantly in microtissues consisting of A549 cancer cells (A3 + A5). Both cancer cell line monocultures as well as Colo699 co-cultures showed no signs for hypoxia. Bar: 100 µm.

CA IX was significantly expressed in microtissues that consisted of A549 cancer cells co-cultured with fibroblasts alone or together with endothelial cells. However, no CA IX expression was found in cancer cell monocultures or in Colo699 co- and tri-cultures after ten days of incubation (Fig. 8).

### Secretion of angiogenic related growth hormones and cytokines

To examine whether microtissues secrete pro-angiogenic factors, supernatants from monocultures (A549, Colo699, SV80) and co-cultures (A549/SV80, Colo699/SV80) were harvested on the day endothelial cells were added, and analysed using a multiplex immunoassay which is based on the luminex technology (Fig. 3A,B). In this study, we focused on VEGF-A, FGF-2, PDGF-BB and VEGF-D expression. In our system VEGF-A and FGF-2 and PDGF-BB could be detected, while VEGF-D was not measurable. In monocultures, the fibroblast cell line SV80 secreted the highest amount of both VEGF-A (mean: 4200 pg/mL) and FGF-2 (mean: 71 pg/mL), respectively. While A549 monocultures secreted VEGF-A in high amounts (mean: 1800 pg/mL), FGF-2 could only be detected in low concentrations (mean: 50 pg/mL). In supernatants harvested from Colo699 monocultures also both factors were detectable, but only at low concentrations (VEGF-A mean: 350 pg/mL, FGF-2 mean: 40 pg/mL).

Therefore, in general co-cultures of cancer cells with either fibroblasts or endothelial cells delivered the highest amounts of pro-angiogenic factors. Whereas, co-cultures consisting of A549/SV80 cells secreted the highest amounts of both cytokines (VEGF-A mean: 8600 pg/mL, FGF-2 mean: 168 pg/mL) compared to Colo699/SV80 microtissues (VEGF-A mean: 3600 pg/mL, FGF-2 mean: 70 pg/mL).

PDGF-BB was only secreted significantly into the supernatant after ten days of incubation when microtissues consisted of both cancer cells and endothelial cells. Higher concentrations of PDGF-BB were observed in both co-cultures with the primary endothelial cell line L-HMVEC (PDGF-BB mean: A549/L-HMVEC - 59,6 pg/ml, Colo699/L-HMVEC – 42,1 pg/ml) in contrast to HUVEC containing co-cultures ((PDGF-BB mean: A549/HUVEC – 10,9 pg/ml, Colo699/HUVEC – 16,1 pg/ml). When fibroblasts were added, PDGF-BB amounts decreased significantly in the supernatant (Supplementary Fig. 5).

### Inhibition of endothelial cell migration and formation by anti angiogenic drugs

To evaluate if the migration of endothelial cells can be blocked, inhibitors of pro-angiogenic factors, bevacizumab and nindetanib, were applied on microtissues.

After incubation of microtissues with two different dose of either bavicizumab or nindetanib for 48 hours their viability and migration into the microtissues was displayed either by flow cytometry or IHC. Flow cytometry data of bevacizumab incubated microtissues are not shown because there was no significant difference observed to the nindetanib data.

In microtissues without drug incubation, within five days endothelial cells migrated into the inner core of the microtissues, aggregating themselves as mentioned above next to fibroblasts (Fig. 3D). An incubation with the two substances led to a decrease in migration of endothelial cells into microtissues. Endothelial cells remained at the outer part of the spheroids either only adherent to the surface of the microtissue or in the areas just below the outer rim of epithelial E-cadherin positive cells.

These results were observed in all microtissues either containing A549 or Colo699 cancer cell lines and with both anti angiogenic drugs. A dose depending effect on the inhibition of endothelial cell migration was observed (Fig. 3E).

These results were proven by the analyses of nindetanib and bevacizumab incubated microtissues by FACS. Here significant decreased numbers of CD31 positive cells were found in lysed microtissues after drug incubation compared to the control group indicating an inhibition of migration and adherence to tumour/fibroblast microtissues (Fig. 3D).

## Discussion

We describe a novel multicellular 3D cell culture model using not only two different non-small cell lung cancer (NSCLC) cell lines together with a lung fibroblast cell line but also in combination with two different endothelial cell lines in a hanging drop technology.

So far, 3D cell culture models that mimic endothelial cell behaviour, angiogenesis and drug interactions are rare and have never been performed in a hanging drop model^12^.

In our model, within five days, endothelial cells migrated into the microtissues and located themselves predominantly in the neighbourhood of fibroblasts. We demonstrate that the migration of endothelial cells depend on the presence of pro-angiogenic factors such as VEGF and FGF, being produced during the incubation period.

Furthermore, we highlight the crucial role of fibroblasts regarding the generation and secretion of cytokines and growth factors into the supernatant. Fibroblasts generate a gradient of angiogenic stimulators, decreasing from the rim towards the core of the microtissue. Thus, endothelial cells migrate according to this gradient of stimulators, which is similar to a chemotaxis assay such as in a Boyden chamber^17, 18^.

The architecture of our microtissues and the aggregation of fibroblasts underline the fact that fibroblasts are the leading cells in producing the angiogenic factors. In contrast to FGF and VEGF, PDGF-BB amounts, however, were found to be significantly lower in supernatants of tri-cultures containing the fibroblast cell line SV80. PDGF-BB is known as a major driver of fibroblast growth and regeneration and thus was consumed by SV80 cells during microtissue formation^19, 20^.

It is known that hypoxia plays an essential role in triggering cytokine secretion and thus supports endothelial cell migration. In microtissues, hypoxia is induced by oxygen deprivation starting at a diameter of 200–250 µm^21^. We clearly show that in our model hypoxia is playing a crucial role, especially in A549 containing microtissues where stromal cells are included. In these microtissues by production of ECM cell-cell contacts are increased and thereby less oxygen was able to perfuse to the core of the spheroids. Interestingly, Collagen VI was also expressed significantly only in our microtissues that included stromal cells. It is known that Collagen VI also indicates a more aggressive phenotype in cancers and is associated with a worse prognosis for patients^22^.

Nevertheless, hypoxia is increasing towards the inner core of the microtissues (size varying between 250 µm to 500 µm) and therefore stresses cells that are predominantly located at this place. We show, that fibroblasts built up the inner parts and are thereby mostly affected by oxygen deprivation stimulating the secretion of angiogenic cytokines^23, 24^.

In accordance with our data, there are the results of a previously published study where the same NSCLC cell line was combined with fibroblasts and endothelial cells by PEGylated-fibrin hydrogels^12^. Instead to their linear method of seeding cells, we present here data also for a consecutive approach (Fig. 1). Therefore, their study has its limitations. Migration of endothelial cells into a cancer cell containing microtissue cannot be mimicked with the hydrogel assay because endothelial cell aggregation is not induced by a migration towards an increasing cytokine gradient but by direct cell-cell interaction only not as in our model.

In accordance to our results, changes in protein expression in co-cultures with endothelial cells suggest a transformation of tumour cells to a more mesenchymal and aggressive phenotype, which is indicated by ASMA expression/upregulation in all cells and morphology alterations of A549 cells resembling so called signet ring cells). In addition, fibroblasts also started to resemble a so-called cancer associated fibroblast phenotype by expressing ASMA^12, 25^.

The expression of ASMA define epithelial to mesenchymal transition (EMT) of cancer cells as a result of complex cancer – stroma cell interactions was recently published^25, 26^. It was shown that breast cancer cell lines start to express ASMA when co-incubated with endothelial cells in a transwell co-culture system. This transformation was not induced by direct cell to cell contact, but by secretion of hepatocyte growth factor (HGF) of endothelial cells into the supernatant. These findings are in accordance with our results, where both cancer cells and fibroblasts started to express ASMA as soon as they were co-incubated with endothelial cells. We have previously demonstrated that without adding endothelial cells to microtissues, ASMA expression was only induced in co-cultures of A549 cancer cells with SV80 fibroblasts but not in combination with Colo699^11^.

Based on the secretion of angiogenic factors, we have chosen two different antiangiogenic drugs to be tested in our system. First, bevacizumab as a specific inhibitor for VEGF which is applied for the treatment of a variety of malignant disorders and furthermore, nindetanib was chosen as tyrosine kinase inhibitor (TKI) targeting multiple receptors of pro-angiogenic factors (FGF, PDGF and VEGF) which were in part significantly secreted in our model^27–29^.

While both drugs inhibited the invasion of endothelial cells, only nindetanib could completely block the adherence of endothelial cells to the microtissue.

This inhibiting effect of nindetanib in comparison to bevacizumab can be explained by the fact that nindetanib targets multiple receptors that play a crucial role in angiogenesis.

*In vitro* models where angiogenic drugs can be screened in a functional way are rare. So far, the best studied model for testing nindetanib has been published for idiopathic fibrosis but not for malignant tumours^29^.

We demonstrate that our model is a promising tool for the generation of tumour spheroid co-cultures with the option of endothelial cell implementation. Furthermore, we established a model where angiogenesis and especially the migration ability of endothelial cells into the tumour can be mimicked without the need of cytokine addition or artificial ECM substrates. Therefore, this model can be implemented for drug testing where not only cellular cytotoxicity can be analysed but also functional effects on the behaviour of cells.

## Material and Methods

### Cell culture

The NSCLC cell lines A549 and Colo699 (DSMZ, ACC107, ACC196), the lung fibroblast cell line SV-80 (CLS, 300345), the primary human microvascular endothelial cell line of the lung L-HMVEC (Lonza, CC-2527) and the human umbilical vein endothelial cell line (Lonza, CC-2935) were used for our experiments. For 2D culture, cancer cell lines, fibroblasts and HUVECs were cultured as monolayer in DMEM low glucose (PAA, Pasching, Austria) supplemented with 10% FCS (Sigma-Aldrich, Munich, Germany, Lot 010M3396) and 100 U/ml penicillin,100 µg/ml streptomycin solution and 2 mM L-Glutamine (PAA). For primary endothelial cell lines the suggested medium of the manufacturer was used (Lonza, CC-3202). Cells were cultivated at 37 °C in a humidified 5% CO_2_-containing atmosphere.

### 3D cell culture

For the production of 3D mono- and co-cultures, the GravityPLUS^TM^ microtissue culture system (InSphero AG, Zürich, Switzerland) was used. The protocol was previously published by our working group^11^.

In addition, the ratio between tumour cells and endothelial cells was defined as 1:1 (exact numbers: 1250 tumour cells, 1250 endothelial cells and 2500 fibroblasts). The endothelial cells were seeded in two ways into the hanging drops (Fig. 1). In one case they were added as a mixture of single cells together with fibroblasts and tumour cells in the above mentioned ratio on day one. In the other case they were added to pre-incubated co-cultures of tumour cells and fibroblasts on day five. The same number of endothelial cells (1250) was added at this time point to the existing microtissues.

### Flow-cytometry – Viability

Eight microtissues, harvested in 96-well plates were processed for analyses by flow cytometry as already published^11, 13^.

Due to CFSE incubation of endothelial cells, APC labelled Annexin V (BD Pharmingen, 550475) was applied for viability measurements.

Finally, cells were immediately analysed on a BD FACS Calibur. Each data measurement was made up from three pooled microtissues and all analyses were done in triplicates. The whole experiment was repeated three times.

### CFSE labeling of endothelial cells

For the fluorescent labeling of endothelial cells the CellTrace^TM^ CFSE Cell Proliferation Kit (Molecular Probes, Invitrogen) was used. Cells were dissolved and adjusted to 1 × 10^6^ in 1 ml of PBS/0,1% bovine serum albumin (BSA). CFSE solution was added to achieve a final working concentration of 10 µM. Cells were then incubated for 10 minutes at 37 °C. Afterwards, the staining was quenched with five volumes of ice cold cell culture medium and incubated for 5 minutes on ice. Then cells were washed three times with cell culture medium. Labeled endothelial cells were seeded as co-cultures with carcinoma cells and fibroblasts as described.

### Immunohistochemical analyses

#### Sample preparation

Microtissues were rinsed in PBS and then immediately fixed by immersion in cold 4% paraformaldehyde (PFA) in PBS, for four hours at room temperature. Microtissues were then rinsed in PBS again and embedded in 2% agarose (Invitrogen). Excess agarose was removed with a scalpel, and the agarose blocks containing the microtissues were dehydrated in graded alcohols and embedded in paraffin wax (Paraplast regular, Sigma-Aldrich, St. Louis, MO, USA). A Microm ERGO Star Rotary microtome (Microm, Walldorf, Germany) was used to take serial sections of 4 µm thickness. A three to four series of paraffinized sections were then mounted in a meandering pattern on SuperFrost®Plus slides (Menzel-Gläser, Braunschweig, Germany) and dried overnight, then baked at 60 °C for one hour to adhere the sections firmly to the slides. For cyto-architectural orientation, every tenth slide was hematoxylin/eosin stained (HE) using a Shandon Varistain® 24–4 Slide Stainer (Histocom Vienna, Austria).

#### Antisera

Hosts, dilutions, incubation times and sources of primary antibodies as well as heat-induced epitope retrieval (HIER) are listed in Supplementary Table 1.

#### Immunocytochemistry

Immunocytochemistry was carried out on 4 µm sections of paraffin-embedded microtissues in a Ventana Roche Discovery Immunostainer (Mannheim, Germany) according to the DAB-MAP discovery research standard procedure. If required, antigen retrieval was initiated by heat-induced unmasking of the epitopes while the slides were immersed in accordance with the manufacturer’s instructions (short, mild or standard for different incubation times) in EDTA buffer (Cell Conditioning Solution CC1, Ventana). After incubation of the sections with the primary antibodies (listed in Supplementary Table 1) at 37 °C, a biotinylated immunoglobulin cocktail of goat anti-mouse IgG, goat anti-mouse IgM, goat anti-rabbit IgG and protein block (Discovery Universal Antibody, Ventana) was applied for 30 minutes at room temperature. The detection was achieved using the DAB-MAP Detection Kit (Ventana) according to the diaminobenzidine (DAB) development method. Sections were finally counterstained with hematoxylin (Ventana) for four minutes. Subsequently, sections were manually dehydrated in downgraded alcohol series, cleared in xylene and cover slipped permanently with Entellan^®^ (Merck, Darmstadt, Germany).

Digital images of HE- and immunostained slides were acquired in AxioVision microscope software linked to an AxioCam HRc color camera and an AxioPlan 2 microscope (Zeiss, Jena, Germany).

Ki-67 positivity was determined by counting Ki-67 positive and negative cell nuclei in three different representative microtissues. Thereafter, the percentage of positive cell nuclei to whole cell number was calculated. Mean and standard deviation and statistical significance was calculated as described before.

### Pro-angiogenic factors profiling of supernatants– Multiplex Immunoassay

To analyse supernatants of microtissues for pro-angiogenic factors the “ProcartaPlex Human Growth Factor Panel 11 plex” – immunoassay (Affymetrix-eBioscience, Germany) that refers to the luminex technology, was performed according to the manufacturer’s instructions. In brief, supernatants of 16 single spheroids per approach were harvested and pooled on day 5 after starting 3D cell cultures. To remove remaining cells, pooled supernatants were centrifuged at 400 g at 4 °C for 5 minutes, subsequent a second centrifugation step was performed at 10 000 g at 4 °C for 5 minutes to remove cell debris. After centrifugation, all samples were snap-frozen in liquid nitrogen and stored at −80 °C until use. After thawing on ice, samples (50 µL) in duplicate were added to a 96-well flat bottom plate with a panel of anti-proangiogenic factors antibodies covalently linked to magnetic beads. Standards (7-points dilutions) and controls were added at this time. The plate was incubated for 30 minutes at room temperature in the dark while shaking at 400 rpm, subsequent the plate was further incubated overnight at 4 °C in the dark without shaking. After overnight incubation, the plate was shaken for further 60 minutes at room temperature at 400 rpm and then washed twice. In the next step 25 µL of detection antibody mixture were added to each well and the plate was incubated, protected from light, for 30 minutes on a plate shaker at room temperature at 400 rpm. Afterwards, the plate was washed twice again and 50 µL of a streptavidin-PE (SAPE) solution were applied to each well. Incubation was performed to same conditions as in the step before. Before measuring, the plate was washed twice and 120 µL reading buffer were added to each well and incubated on a plate shaker at 500 rpm for 5 minutes at room temperature. Samples were measured using a Magpix (Luminex) and data were analysed by the “ProcartaPlex Analyst 1.0” software. For this study we analysed VEGF, FGF-2, PDGF-BB and VEGF-D.

### Migration of endothelial cells

Endothelial cells were labelled with CFSE and added to microtissues as described above. Every 24 hours an image was taken by an epifluorescence microscope (Leica DMi8). Images were processed with the Leica software LAS X.

### Cytotoxicity of anti-angiogenic drugs

To evaluate cytotoxic effects of nintedanib (Selleckchem, USA), cells, used in the experiment, were seeded in 24-well plates at a cell number of 1 × 10^4^/well. Twenty-four hours after seeding, cells were treated with nintedanib in triplicates at following concentration [nM]: 0, 25, 50, 100, 250 and 500. Treated cells were analysed after 24 hours of incubation for apoptotic and late apoptotic/necrotic cells by means of annexin V-FITC/propidium iodide staining. Stained samples were measured using a Cytometer FC 500 (Beckmann Coulter, Germany) and gained data was analysed by the use of FlowJo 10.0.7.

### Inhibition of migrating endothelial cells

To investigate whether the migration of endothelial cells into microtissues can be blocked, two anti-angiogenic agents, the triple angiokinase inhibitor nintedanib (Selleckchem, USA) and the monoclonal anti-VEGF antibody bevacizumab (Roche, Germany), were applied to the system. To perform these experiments, 3D mono- and co-cultures of A549 cells were cultivated as mentioned in the 3D cell culture section. Five days after starting the 3D cell culture, endothelial cells were added to formed microtissues with one of the two anti-angiogenic substances. To controls, only endothelial cells were applied. Concentrations used in this study, were for nintedanib 50 and 100 nanomolar [nM], and for bevacizumab 0.1 and 1 mg/mL. Two days after incubation, microtissues were harvested and prepared either for flow cytometry or for immunohistochemistry, to be analysed for CD31+ cells. For immunohistochemistry analysis, microtissues were prepared as mentioned in the immunohistochemical analyses section. To analyse microtissues in the flow cytometer for infiltrated endothelial cells they were handled as mentioned in the flow cytometer section. Endothelial cells were identified by using a FITC labelled monoclonal antibody against the surface marker CD31 (Beckman Coulter, Germany). Stained samples were measured using a Cytometer FC 500 (Beckmann Coulter, Germany) and gained data was analysed by use of FlowJo 10.0.7.

## Electronic supplementary material

Supplementary Information
