# Supplementary material for: Development of a 3D angiogenesis model to study tumour – endothelial cell interactions and the effects of anti-angiogenic drugs
Source: Sci Rep. 2017 Jun 7;7:2963. doi: 10.1038/s41598-017-03010-6 (PMC5462801; doi:10.1038/s41598-017-03010-6)
Supplement: Supplementary file 1 — Supplementary Information [file 41598_2017_3010_MOESM1_ESM.pdf]

Development of a 3D angiogenesis model to study tumour – endothelial cell interactions and the effects of anti-angiogenic drugs

Arno Amann[1], Marit Zwierzina[2] Stefan Koeck[1] Gabriele Gamerith[1], Elisabeth Pechriggl[3], Julia M. Huber[1], Edith Lorenz[1], Jens M. Kelm[4], Wolfgang Hilbe[5], Heinz Zwierzina[1], Johann Kern[6]

[1] Medical University of Innsbruck, Department of Internal Medicine V, Anichstraße 35, 6020 Innsbruck, Austria

[2] Medical University of Innsbruck, Department of Plastic, Reconstructive and Aesthetic surgery, Anichstraße 35, 6020 Innsbruck, Austria

[3] Medical University of Innsbruck, Division of Clinical and Functional Anatomy, Department of Anatomy, Histology and Embryology, Müllerstraße 59, 6020 Innsbruck, Austria

[4]InSphero AG, Wagistrasse 27, CH-8952 Schlieren, Switzerland

[5]Wilhelminenspital, Medical Department, Centre for Oncology, Haematology and Palliative care, Motlearstraße 37, 1160 Vienna, Austria

[6]Tyrolean Cancer Research Institute, Innrain 66, 6020 Innsbruck, Austria

Corresponding author:

Arno Amann, M.D

Laboratory for Translational Cancer Research

Department of Internal Medicine V (Haematology and Oncology)

Medical University Innsbruck

Anichstrasse 35, A-6020 Innsbruck, Austria

Phone: ++43/512/504/83094

E-mail: arno.amann@tirol-kliniken.at

Supplementary Table 1. Primary antibodies used for immunohistochemistry.

Antibody	Host	Dilution	Antigen unmasking	Incubation time	Source (# number)
**Anti-E-cadherin**	mouse mAB	ready-to-use	CC1 standard	60 min	Novocastra (E601)
**Anti-Vimentin**	mouse mAB	ready-to-use	CC1 short	60 min	Linaris (E034)
**ASMA**	mouse mAB	ready-to-use	none	12 min	Linaris (E046)
**Anti-Ki-67**	rabbit mAB	ready-to-use	CC1 standard	60 min	Ventana (790-4286)
**Anti-CD31**	mouse mAB	ready-to-use	CC1 mild	60 min	Covance (SIG – 3632-26)
**Anti-vWF**	rabbit pAB	ready-to-use	CC1 standard	32 min	Cell Marque (760-2642)
**Anti-Carbonic Anhydrase IX antibody**	rabbit pAB	ready-to-use	CC1 standard		Abcam (ab15086)
**Anti-Collagen VI antibody**	rabbit pAB	ready-to-use	CC1 standard		Abcam (ab6588)

Supplementary Table 2: Summary of IHC protein expression pattern. +/- displays the presence/absence of the indicated protein.

Cell types included in microtissues	**A549+SV80+ HUVEC**		**A549+SV80+L-HMVEC**		**Colo699+SV80+ HUVEC**		**Colo699+SV80+ L-HMVEC**		**SV80+HUVEC/ L-HMVEC**	
Days	**5**	**10**	**5**	**10**	**5**	**10**	**5**	**10**	**5**	**10**
**E-cadherin**	+	+	+	+	-	-	-	-	-	-
**Vimentin**	+	+	+	+	+	+	+	+	+	+
**α-SMA**	+	+	+	+	+	+	+	+	+	+
**Ki-67**	+	+	+	+	+	+	+	+	+	+
**CD31**	+	+	+	+	+	+	-	-	+	+
**vWF**	+	+	+	+	+	+	+	+	+	+
**Collagen VI**	+	+	+	+	+	+	+	+	+	+
**Ca IX**	+	+	+	+	-	-	-	-	-	-

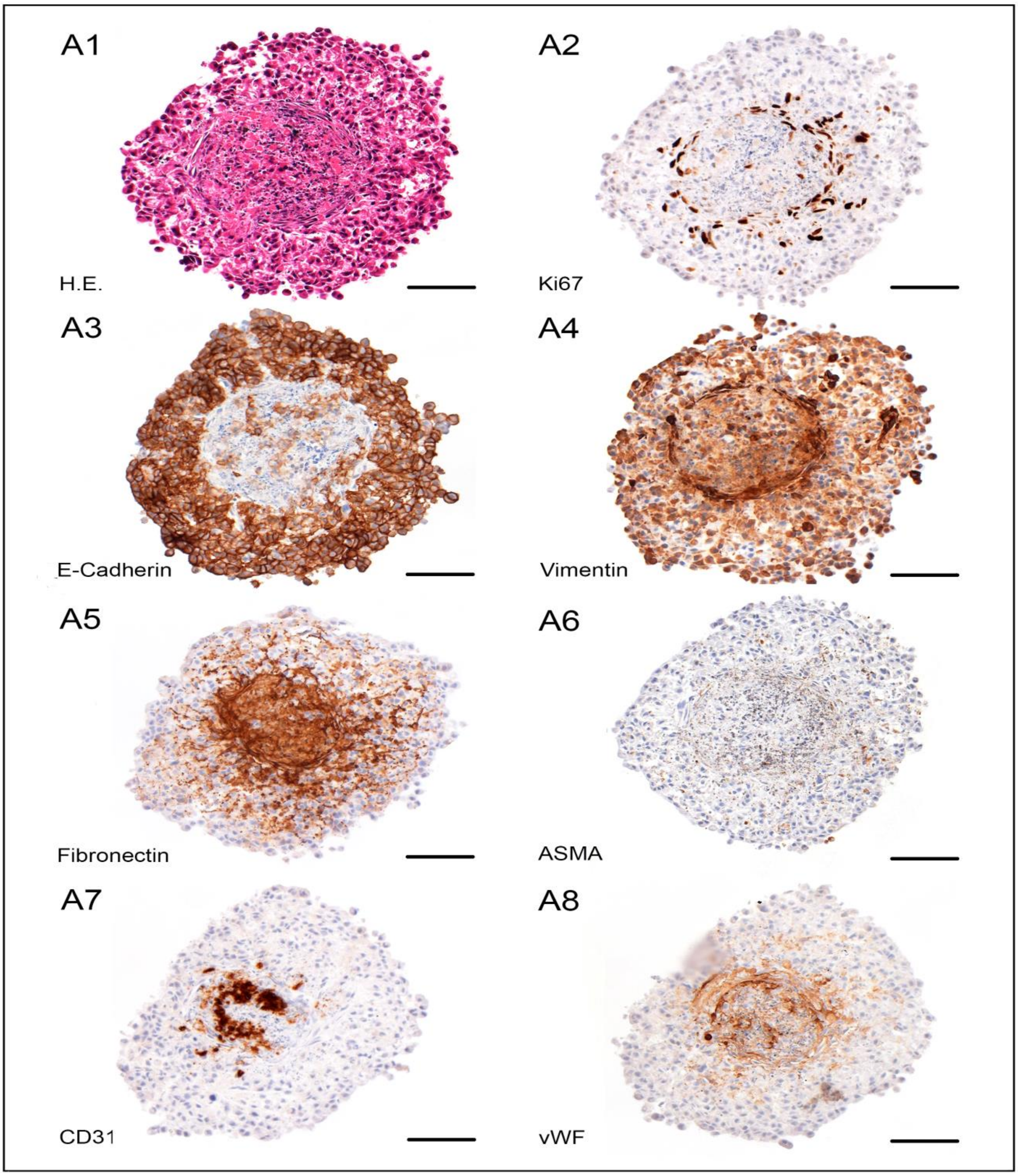

Supplementary Figure 1:

A549 tri-cultures microtissue protein expression pattern (A1-A8): IHC slices of A549 with SV80 and L-HMVEC after 10 days. Endothelial cells are located in the fibroblast core of microtissues (A7). All cells started to express alpha-smooth muscle actin (A7) and vimentin (A4). Bar: 100 µm. Inlet showing formation of endothelial cells as coherent structure. Bar: 100 µm

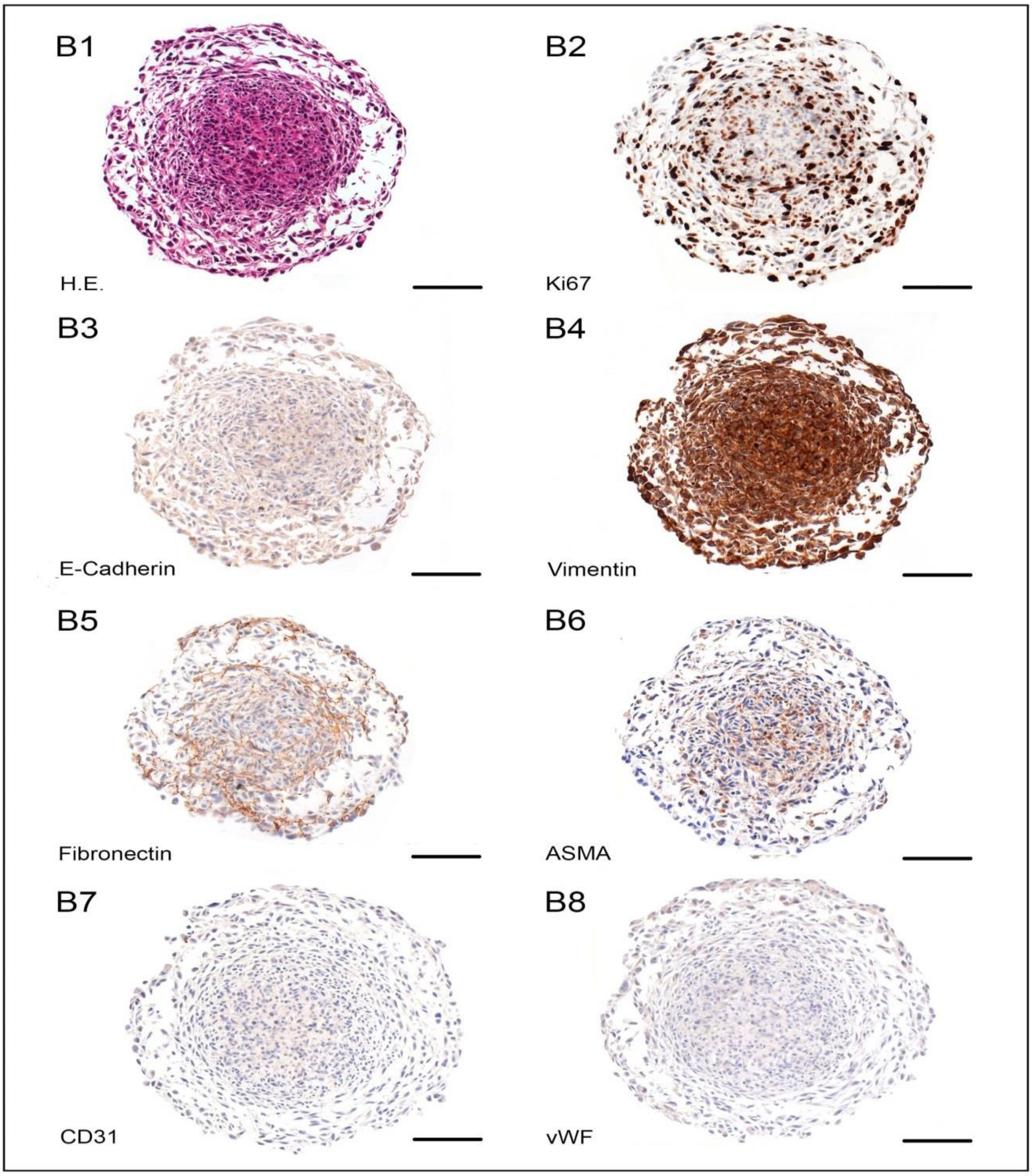

Supplementary Figure 2:

Colo699 tri-cultures microtissue protein expression pattern (B1-B8): IHC slices of Colo699 with SV80 and L-HMVECS after 10 days. No endothelial cells were detected after ten days of incubation (B7). All cells expressed ASMA (B6), vimentin (B4) and no E-cadherin (B3). Bar: 100 μm.

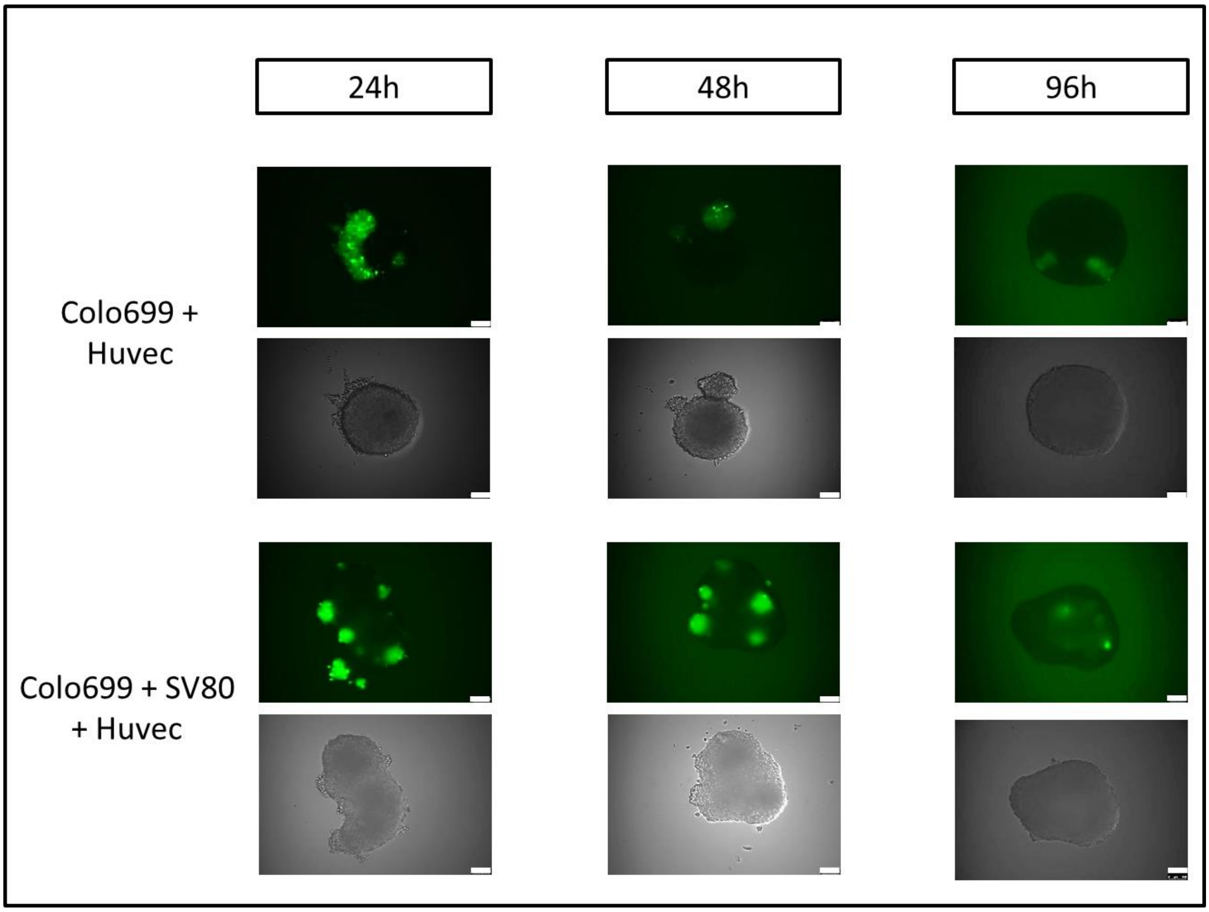

Supplementary Figure 3: Migration of endothelial cells in Colo699 containing microtissues: Endothelial cells were labelled with CFSE and tracked by epifluorescence every day for 96h. Cells attached themselves after 24h to the microtissue and migrated during cultivation time to the core of the spheroids. Bar: 75 µm.

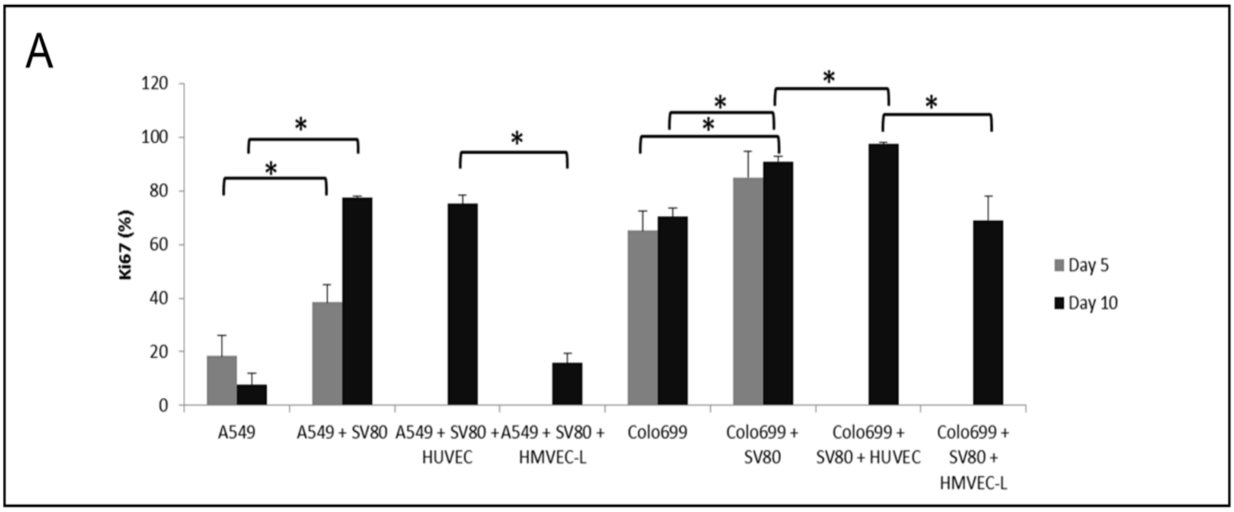

Supplementary Figure 4: Cell proliferation and activation pattern: Mean and standard deviation positivity was calculated by counting Ki67 positive and negative cell nuclei on slices of three different representative spheroids. Thereafter, the percentage of positive cell nuclei to whole cell cumber was calculated. Co-cultivation of both tumour cell lines with the human microvascular cell line of the lung (L-HMVEC) led to a significant downregulation ($p<0,001$) of Ki67 expression in all microtissues. In contrast, the co-incubation of Colo699/SV80 co-cultures with the human umbilical vein endothelial cell lines (HUVEC) led to a significant higher ($p>0,05$) Ki-67 positivity of cells.

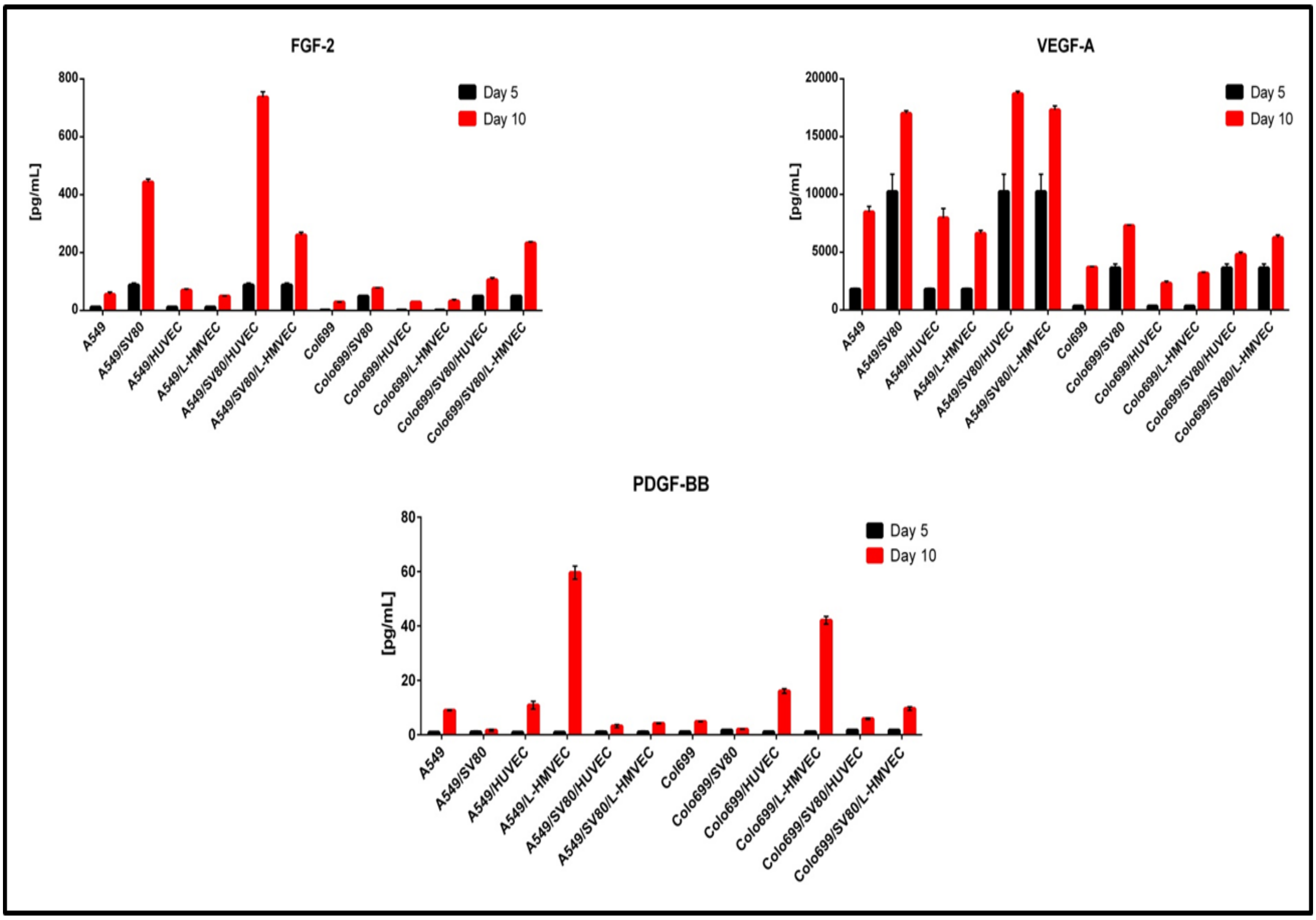

Supplementary Figure 5: Expression of angiogenic factors (VEGF-A, FGF, PDGF-BB): In our system VEGF-A and FGF-2 and PDGF-BB could be detected, while VEGF-D was not measurable. In general co-cultures of cancer cells with either fibroblasts or endothelial ells delivered the highest amounts of pro-angiogenic factors (FGF, VEGF-A). Whereas, co-cultures consisting of A549/SV80 cells secreted the highest amounts of both, compared to Colo699/SV80 microtissues. PDGF-BB was only secreted significantly when microtissues consisted beside cancer cells also of endothelial cells after ten days of incubation. Higher concentrations of PDGF-BB were observed in both co-cultures with the primary endothelial cell line L-HMVEC in contrast to Huvec containing co-cultures. When fibroblasts were added, PDGF-BB amounts in the supernatant decreased significantly.
